# Comparative transcriptomics analysis of compatible wild type and incompatible Δ*laeA* mutant strains of *Epichloë festucae* in association with perennial ryegrass

**DOI:** 10.1016/j.dib.2019.103843

**Published:** 2019-03-16

**Authors:** M. Rahnama, P. Maclean, D.J. Fleetwood, R.D. Johnson

**Affiliations:** a AgResearch, Grasslands Research Centre, Palmerston North, New Zealand; b School of Biological Sciences, University of Auckland, New Zealand; c Biotelliga Ltd, Auckland, New Zealand

## Abstract

*Epichloë festucae* fungi form bioprotective endophytic symbioses with perennial ryegrass. Although this interaction is economically important, relatively little is known about the molecular processes and regulatory genes that are involved in establishing a compatible symbiosis. The present study utilised next-generation sequencing to investigate the genes required for establishing a compatible symbiotic interaction between *E. festucae* and perennial ryegrass. A comparative transcriptomics study, comparing the compatible symbiotic interaction of *E. festucae*/perennial ryegrass with the incompatible interaction of a Δ*laeA* mutant strain of *E.festucae*/perennial ryegrass, was performed two weeks after inoculation. Differentially expressed genes were identified and classified according to gene ontology and functional annotation analyses. The raw data of this study have been deposited at SRA database with the BioProject ID PRJNA513830.

**Table:** Specifications table

Subject area	Biology
More specific subject area	Molecular Plant-Microbe Interactions, transcriptomics
Type of data	Transcriptome sequences, table, graph
How data was acquired	IlluminaHiSeq™ 4000
Data format	Raw (Fastq) sequences
Experimental factors	*L. perenne* seedlings inoculated with two strains of *E. festucae* for two weeks under 16 h of light and 8 h dark
Experimental features	Comparative transcriptomic analyses of *L. perenne* inoculated with wild type and Δ*laeA* mutant strains of *E. festucae*
Data source location	Auckland, New Zealand
Data accessibility	Data can be accessed from NCBI SRA (Bioproject ID: PRJNA513830) (https://www.ncbi.nlm.nih.gov/sra/PRJNA513830)

**Table:** 

**Value of the data** • Transcriptomics data from the E. festucae/perennial ryegrass interaction was obtained and this is the first such study specifically looking at an early time point during the association. • Comparative transcriptomics of a compatible E. festucae wild type strain versus an incompatible ΔlaeA mutant strain identified differentially expressed genes important for the early establishment of this symbiosis with perennial ryegrass, and highlighted the role of the laeA gene during this interaction. • Further analysis of the presented data can help to understand the molecular processes important to establish a compatible interaction between E. festucae and perennial ryegrass.

## 1 Data

Data reported here describes the results of a comparative transcriptomics study between compatible symbiotic interactions of *Epichloë festucae*/perennial ryegrass and incompatible interactions of Δ*laeA* mutant strains of *E.festucae*/perennial ryegrass [1]. HiSeq Illumina sequencing included 12 raw sequence data sets that have been deposited into the NCBI SRA database and can be accessed with the Bio Project accession number PRJNA513830. Differentially expressed genes (DEGs) were identified and further analysed using Gene Ontology (GO) (Supplementary Fig. 1A–C) and functional annotation (Supplementary Table 1).

## 2 Experimental design, materials and methods

### 2.1 Sample preparation and RNA isolation

Wild type and Δ*laeA* mutant strains of *E.festucae* were grown on 2.4% potato dextrose (PD, Difco Laboratories) plus 1.5% agar (PD, Difco Laboratories). 7–10 d old endophyte-free seedlings of perennial ryegrass (*L. perenne* ‘Nui’) were inoculated with wild type and Δ*laeA* mutant, generated with a split-marker deletion method [2], strains of *E.festucae* as previously described [3].

Inoculated seedlings were grown for two weeks under 16 h of 650 W/m2 light and 8 h dark. Seedlings were frozen in liquid nitrogen and samples from 4 cm upwards and 0.5cm downwards from the meristem were collected for RNA extraction. 100 seedlings for each sample were pooled in three replicates for each treatment. RNA quality and quantity were determined using an Agilent 2100 Bioanalyzer (Agilent Technologies), Nanodrop Lite spectrophotometer (Thermo scientific) and running on a 1% agarose gel.

### 2.2 RNA-seq (HiSeq) analysis

RNA samples on dry ice were sent to the Beijing Genomics Institute (BGI, Hong Kong) for sequencing and 2 μg of RNA sample used to prepare libraries by BGI standard methodology (http://www.bgi.com/services/genomics/rna-seqtranscriptome/#tab-id-2). Samples were sequenced in two lanes of an Illumina HiSeq4000 (paired end, 100-bp reads) (Table 1).

**Table 1:** General description of RNA seqencing results of two weeks old perennial ryegrass seedlings inoculated with Δ*laeA* mutant strains *Epichloë festucae* before and after mapping analysis.

	*Epichloë festucae*	Δ*laeA* mutant *Epichloë festucae*
Total read numberrowhead	245807532	245109422
Number of total reads after quality trimmingrowhead	245494396	244879692
Percentage of total reads after quality trimmingrowhead	99.87	99.91
Number of mapped readsrowhead	209667702	206242328
Proportion of mapped reads (% of trimmed reads)rowhead	85.41	84.22
Number of reads mapped to Endophyte genomerowhead	16744016	16706144
Percentage of mapped fungal reads to total mapped readsrowhead	7.99	8.10

### 2.3 HiSeq results analysis

Official gene sets of *E. festucae* Fl1 (downloaded from http://csbio-l.csr.uky.edu/m3/draft/m3-v8-2014-12-12.fasta) were mapped against the official genome scaffold (downloaded from http://csbio-l.csr.uky.edu/ef894-2011/gbrowse/ef/) with Exonerate version 2.2.0 using the –est2genome model and keeping alignments scoring at least 50% of the maximal score for each query. The target GFF option was used for the exon coordinates to be imported into RNA-star to enumerate the genes [4].

The reads were trimmed using flexbar version 2.4 [5] and mapped against the prepared database using RNA-star version 2.5.0c [4] (Table 1). The non-directional counts of uniquely mapped read pairs were summed for each gene and analysed using the EdgeR package version 3.10.5 [6] in the R statistical software environment version 3.2.1. Quasi-likelihood negative binomial generalized linear models were generated from the counts within sample type. Fold changes and p-values were generated using Exact Tests for differences between two groups of Negative-Binomial Counts. Of total 8547 genes in *E. festucae* 216 genes were differentially expressed (with two-fold or more difference and an FDR equal to or less than 0.05).

### 2.4 Gene ontology (GO) analysis

Transcript sequences for *E. festucae* were searched against the NCBI nr nucleotide database with an e-value cut off of 1E-5 with the top 10 hits being kept. The xml output, along with the corresponding InterProScan output was run though the Blast2GO Pipeline Version 2.5.0 using all the default settings. A non-redundant list of GO terms was made for each gene (multiple transcripts and proteins deriving from each gene were collapsed into a single gene). This non-redundant list was used in GOEAST and AgriGO test for the enrichment of GO terms in differentially expressed gene lists using Fisher exact tests [7], [8] (Supplementary Fig. 1).

### 2.5 Functional annotation

Official protein sequences for endophyte genes were run through InterProScan 5RC4 to find matches against the InterPro protein signature databases using the default settings. The protein sequences were also searched using BLASTP version 2.2.28+ against the entire Swiss-Prot database as well as the fungal division of UniProt and NCBI reference sequence protein plant and fungi subsets with an e-value cut-off of 1E-20. The official transcript sequences were searched against the NCBI reference sequence RNA plant and fungi subsets using BLASTN version 2.2.28+ with an e-value cut-off of 1E-20. In addition, the official protein sequences were also searched using BLASTP version 2.2.28+ against the fungal KEGG genes database with an e-value cut-off of 1E-20 (Supplementary Table 1).

## Funding resources

This research was funded by a grant from the Royal Society of New Zealand Marsden Fund, contract AGR1002 and AgResearch SSIF funding.
