# Supplementary material for: Comparative transcriptomics analysis of compatible wild type and incompatible ΔlaeA mutant strains of Epichloë festucae in association with perennial ryegrass
Source: Data Brief. 2019 Mar 16;24:103843. doi: 10.1016/j.dib.2019.103843 (PMC6484362; doi:10.1016/j.dib.2019.103843)
Supplement: Supplementary file 2 — Multimedia component 2 [file mmc2.docx]

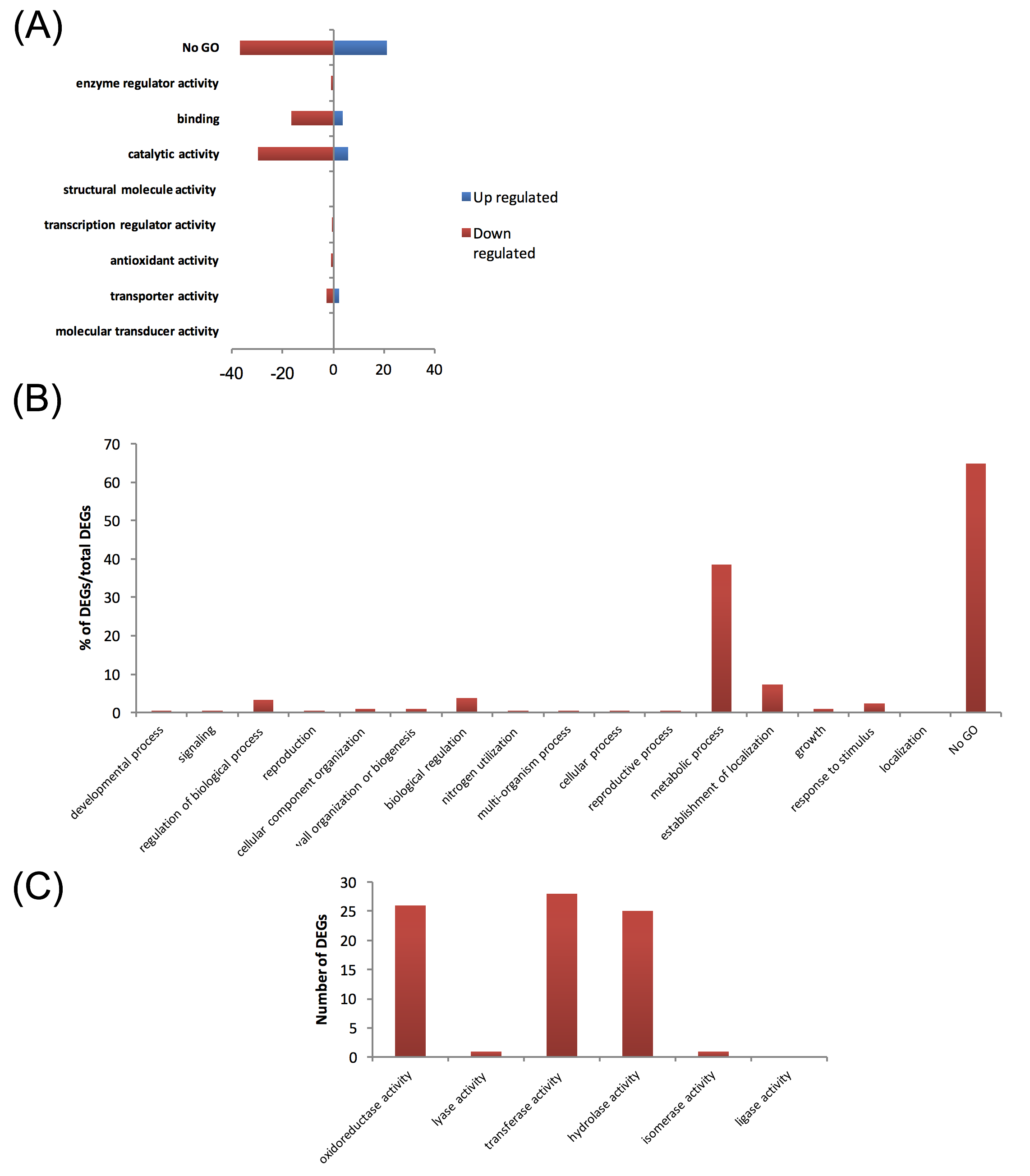


**Supplementary fig. 1-** Differentially expressed genes (DEGs) of Δ*laeA* in comparison with wild type *E. festucae* at two weeks’ post inoculating seedlings classified based on primary ‘Molecular Function’ and ‘Biological Process’ gene ontology (GO) categories. A. Bar charts of organised DEGs based on ‘Molecular Function’ GO category. Categories are as follows: GO:0060089 (molecular transducer activity), GO:0005215 (transporter activity), GO:0016209 (antioxidant activity), GO:0030528 (transcription regulator activity), GO:0005198 (structural molecule activity), GO:0003824(catalytic activity), GO:0005488 (binding), GO:0030234 (enzyme regulator activity). B. Bar chart of organised DEGs based on ‘Biological Process’ GO category. Categories are as follows: GO:0032502 (developmental process), GO:0023052 (signalling), GO:0050789 (regulation of biological process), GO:0000003 (reproduction), GO:0016043 (cellular component organization), GO:0071554 (cell wall organization or biogenesis), GO:0065007 (biological regulation), GO:0019740 (nitrogen utilization), GO:0051704 (multi-organism process), GO:0009987 (cellular process), GO:0022414 (reproductive process), GO:0008152 (metabolic process), GO:0051234 (establishment of localization), GO:0040007 (growth), GO:0050896 (response to stimulus), GO:0051179 ( localization). C. Bar chart showing DEGs organized based on Catalytic activity. Categories are as follows: GO:0016491 (oxidoreductase activity), GO:0016829 (lyase activity), GO:0016740 (transferase activity), GO:0016787 (hydrolase activity), GO:0016853 (isomerase activity), GO:0016874 (ligase activity).
